# Commentary: Personal computers–valuable tools or expensive luxuries?

**DOI:** 10.1155/S1463924681000321

**Published:** 1981

**Authors:** P. B. Stockwell


### Commentary

### Personal computers – valuable tools or expensive luxuries?

Over the past few years a considerable number of companies have developed instruments, both sophisticated and simple, which incorporate microprocessors in their design. Evidence from various conferences and exhibitions seems to suggest that after a number of initial problems several instrument companies are getting the design concepts right and are producing microprocessor-controlled instruments to good specifications and often at a lower cost than previous designs. If in addition the instrument companies can provide a standard output line to allow data and software to be transferred to other devices or computers this will be an added advantage.

However, parallel to these developments, many analytical chemists are attempting to use personal computers in their laboratories, primarily for instrument control and data capture and analysis. Whilst the investment in hardware for these computers is quite low and the development of software an interesting diversion from pure analytical chemistry, it is questionable whether this approach is the best and most economic route. My own view is that the true costs of developing software and interfacing systems to analytical instruments are not fully considered. Developing a program on a computer may be fun, but to design a software system which copes with all parameters including instrument failures, faulty button pushing and the like is difficult and costly. Simply hooking a PET or Apple onto an instrument and mechanising the manual calculation processes previously used is not the best way to automate or computerise the analytical process. A much broader view is required before the detail of the application to a specific instrument is developed. This constitutes a truly analytical approach to the problem; the analytical chemist should not focus attention simply on the measurement process.

### The language problem

Although we have no accepted definition of 'automation' it is true to say that there is also no real understanding of the term 'analysis'. Analysts require to look at the problem in general terms as well as at particular facets of the analysis involved. The analyst must question why he is asked to carry out the tests, to define a suitable strategy for sampling, design his experiments to obtain the maximum data required to the desired degree of accuracy and precision for the overall aims. Many academic groups are considering the problem of correctly defining the terms used in our science and also are creating undergraduate training programmes to meet the specific needs of industry. There are many analysts who are truly analysing problems in the manner described above, but equally as many groups are only looking at a single aspect of the process – whether that be chromatography or spectrophotometry. It is this latter group who are not serving analytical science as well as they might.

### New technology and laboratory manangement

As technology advances one impact on laboratory management is that it becomes increasingly difficult to keep close control over the resources available. Computing is a management tool as well as an analytical tool and the installation of a complete laboratory system will impinge on the management's role considerably. Keeping close control over a laboratory's resources is extremely critical in today's economic environment. Two aspects deserve discussion; firstly education and training, and secondly financial considerations.

### Education

It is important that laboratory managers fully appreciate the needs of their scientists to develop electronic skills, computer skills etc, to cater for the level of instrumentation/automation that is being introduced into their laboratories. The manager may not be able to program a computer himself, but he must be able to evaluate how long a particular software development ought to take and what costs are involved. He must also appreciate the problems of his specialist staff. As requirements for personnel change management must collaborate with academic institutions to provide the necessary training programmes; not only at the undergraduate level but also in post-experience courses devoted to retraining analysts who have long since graduated. It is encouraging that, in addition to the two universities in the UK which are setting up multidisciplinary chemistry or analytical science courses mentioned in a recent issue of this journal, Kings College London is about to offer a chemistry and computing course in the near future. Hopefully the originators of these courses will encourage an industrial input to their programmes and also will not concentrate too greatly on microprocessors. A far broader approach than that previously taken is required and all new technological developments should be integrated into the courses.

Management must assist by helping to define the needs of their organisation, but also they must be willing to encourage senior staff to pass on their skills and expertise to others by allowing them time to lecture at outside courses. The series of post-experience courses at Swansea on Automated Chemical Analysis have been successful in bringing together academics and industrialists to teach the broad aspects of the subject from a theoretical as well as a practical standpoint. Exchange of views between the course staff has forged strong ties and has had the added advantage of bringing together experts from North America, Europe and the UK. One important aim of such a course is to keep up to date and also to respond to individual requirements as much as possible. Managers must keep themselves aware of these post-experience courses and invest sufficient funds in training programmes so that their staff are kept adequately up-dated on the latest developments in analytical chemistry and automation.

### Budget control

During the last decade it became fashionable to invest in expensive instrumentation; computers, mass spectrometers and the like; sums of the order of £50K to £100K for an instrument were not uncommon. However, one of the major costs in a laboratory today is manpower – human resources are not only the most costly budget element but also the most difficult to manage. It is important for managers and staff to be aware of the costs of staff time. This is particularly so for software development. Whenever an instrument is being evaluated for purchase not only should the cost of the initial investment be considered along with maintenance and service support and additional resources but also the time involvement from staff. Often the best approach to solving a problem is to invest more capital money at the outset rather than undercutting the capital budget and then having to devote expensive staff time to upgrade inadequate instrumentation. Management must also take firm positive decisions and only invest in projects which best serve the aims of the laboratory and reject any others. Simply partially funding all programmes to a limited degree will not best serve either the groups working on each programme or the organisation as a whole.

SHIMADZU

High performance
but low cost G.C. and integrators

Ideal for use in research
& quality control laboratories

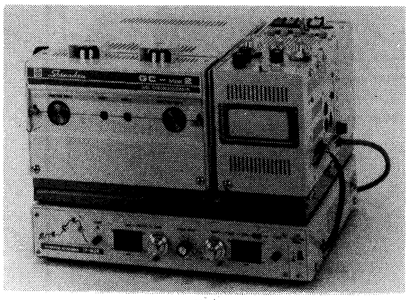

GC MINI 2

- Dual FIDs.
- Wide range amplifier.
- Independent temperature control for oven and injection/detector heaters.
- Full flow controls.

PRICE FROM £2200.
Plus VAT.

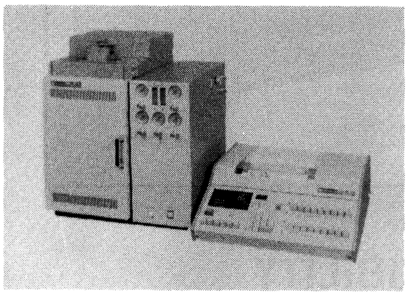

GC RIA SERIES

- Choice of detectors
- Built in computing integrator
- All electronic controls programmed from data processor
- Full flow controls and injector purging system.

PRICE FROM £5600
Plus VAT.

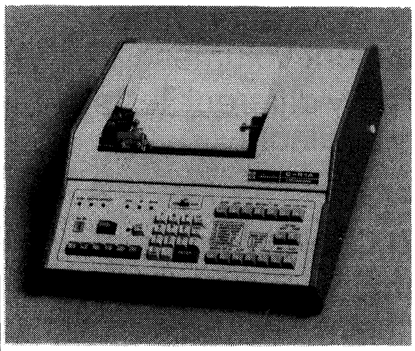

COMPUTING INTEGRATORS
CRIA

- Fully alphanumeric printer plotter.
- Performs all calculations, including 2 point calibration.
- Two month memory protection against power failure.

PRICE £2195 Plus VAT.
CEIB.
Printer version of above
PRICE £1550 Plus VAT.

*Full colour brochures available on request from:—*

DYSON INSTRUMENTS

Sunderland House, Station Road,
Hetton, Houghton Le Spring,
Tyne & Wear, DH5 0AT
Telephone: (0783) 260452, 260433

The area of computer applications is a major area of concern to management and one where close control is required. Teaching chemists to programme a computer can be a great advantage but this knowledge is best used if the chemist uses it to broaden his horizon so that he can precisely specify the requirements for the analysis or instruments to computer specialists who can then develop the software correctly and economically. A chemist may well be able to write a simple programme but tailoring the software to meet such constraints as continuous unattended operation is more difficult and best left for experts in this area.

*P.B. Stockwell*

### Swansea Summer School of Automatic Chemical Analysis 1981

With topics from operational amplifiers to continuous flow analysis and from microprocessors to automated instrumental analysis, this years Summer School at University College, Swansea gave delegates an overview of the entire field of automation in chemical and clinical laboratories. It was a large and complex programme, bringing together experts from many countries to describe their work to over fifty representatives from as many different fields. A total of eighteen lectures were scheduled for the morning sessions, with practical work and tutorials in the afternoons.

The difficult task of reconciling the requirements of such a diverse group was solved quite elegantly. A straw poll was held at the begining of the week to select tutorial topics, and six of these were then scheduled simultaneously. Despite the opportunities for confusion thus offered, the efficient organisation ensured that all delegates had access to the expertise in which they were interested.

In addition, ten instrument companies allowed participants to use their latest products in the practical sessions. When one recalls the advice repeated by many of the experienced lecturers to buy rather than build, these sessions gave invaluable hands-on experience of the available hardware.

Highlights can only be a personal choice. My choice, in no particular order, would include Prof. Gary Horlick from the University of Alberta describing the architecture of microprocessors, and Prof. D.L. Massart from Vrije Universiteit, Brussels showing how to make sense of the mass of data generated by the use of automatic analysis. With the emphasis on data collection and analysis during the week, the timely reminder from Prof. Jack Betteridge that the objective should be to do the chemistry better or in a novel way was needed. Finally, a mention for Dr. Kent Stewart who, not content with the invention of automated multiple flow injection analysis, tentatively suggested that his most recent experiments with interfacing computers with analytical instruments may be the beginning of robotics!

*Dr. J.P. Chester*

